# Is Hospital Hospice Service Associated with Efficient Healthcare Utilization in Deceased Lung Cancer Patients? Hospital Charges at Their End of Life

**DOI:** 10.3390/ijerph192215331

**Published:** 2022-11-20

**Authors:** Dong Jun Kim, Sun Jung Kim

**Affiliations:** 1 Division of Cancer Control and Policy, National Cancer Center, Goyang 10408, Republic of Korea; 2 Department of Health Administration and Management, College of Medical Science, Soonchunhyang University, Asan 31538, Republic of Korea; 3 Center for Healthcare Management Science, Soonchunhyang University, Asan 31538, Republic of Korea; 4 Department of Software Convergence, Soonchunhyang University, Asan 31538, Republic of Korea

**Keywords:** hospice care, lung neoplasms, fees and charges, retrospective studies

## Abstract

In July 2015, South Korea began applying National Health Insurance reimbursement to inpatient hospice service. It is now appropriate and relevant to evaluate how hospice care is associated with healthcare utilization in terminal lung cancer patients. We used nationwide NHI claims data of lung cancer patients from 2008–2018 and identified a sample of patients deceased after July 2016. We transposed the dataset into a retrospective cohort design where a unit of analysis was each lung cancer patients’ healthcare utilization. The differences in hospital charges per day were investigated depending on the patient’s use of hospice service before death with the Generalized Linear Model (GLM) analysis. Additionally, subgroup analysis and the propensity score matching method were used to validate the model using the claims information of 25,099 patients. About 17.0% of patients used hospice services (*N* = 4260). With other variables adjusted, hospice service utilization by deceased lung cancer patients was associated with statistically significant lower hospital charges per day at the end of life (1 month, 3 months, and 6 months before death) compared to non-users. A similar trend was found in the propensity score matching model analysis. We found lower end-of-life hospital charges per day among lung cancer patients who received hospice services near death. The ever-expanding aging population requires health policymakers and the National Health Insurance program to expand hospice services for terminal cancer patients in underserved regions and hospitals that do not provide hospice.

## 1. Introduction

Cancer is a significant cause of death worldwide and continues to be one of the most dreaded modern health threats [1]. Over the past decades, cancer has been the leading cause of death in South Korea. In 2018, there were 28,628 incident lung cancer cases (out of a total of 243,837 cancer cases; 53% male) and 17,852 deaths (out of 79,153 deaths; 74% male) in South Korea [2]. As South Korea enters into an aged society, cancer incidence and deaths will increase, making it imperative that cancers be given systematic management [3]. In addition, traditional and newly developed cancer treatments have a large economic burden [4,5]. Of note, the increase in medical expenses exceeds the rate of inflation and the national budget level [5]. An ideal treatment would be more effective yet less aggressive, but such innovations are typically more expensive [6]. In the United States, 27–30% of the Medicare budget is consumed in the last year of life, with most of these expenses spent on life-sustaining treatments [7]. Many patients spend the majority of their medical bills in the 30 days before they die [8]. The healthcare costs of cancer patients were markedly increased to show a “U” shape at the first stage of diagnosis and the end of life [9,10]. Furthermore, hospital charges are known to increase rapidly during the last months of life; despite some terminal cancer patients preferring to receive treatment and die at home, most patients die in hospitals [10,11,12].

Regarding hospice palliative care, pain is an essential issue for patients with terminal cancers and should be evaluated more comprehensively, rather than regarded as an unpleasant sensation associated with simple tissue damage [13]. The primary cause of cancer pain is tissue damage and nerve stimulation caused by malignant tumors, but the patient’s emotional, social, spiritual, and cultural phenomena are factors that can also influence pain [13]. Hospice palliative care is medical care aimed at improving the quality of life for terminal cancer patients and their families through comprehensive assessment and treatment of the physical, psychosocial, and spiritual areas, including relief of pain and symptoms [14]. The rate of hospice use among all cancer patients who died was 17.5% in 2016 and 20.0% in 2017, up from 7.3% in 2008 [14]. However, this figure was far behind as compared to the United States (52.0%), the United Kingdom (46.6%), Canada (40.8%), and Taiwan (39.0%) in 2011 [15].

Starting from July 2015, South Korea began applying National Health Insurance reimbursement for inpatient hospice services. Using National Health Insurance claims data, it is now appropriate to evaluate how hospice care is associated with healthcare utilization in terminal lung cancer patients [16]. In 2016, 93% of patients and their families who received services from a Korean hospice institution reported very high satisfaction and improved uncomfortable symptoms including nausea, vomiting, and sleep disturbance [17]. In addition, they also received emotional and physical help through various programs such as music and art therapy [18].

Previous research reported that hospice palliative services were associated with lower end-of-life healthcare costs for Medicare patients in the United States [18], and implementation of palliative care and hospice systems reduced hospitalization [19,20,21]. However, research on hospice palliative services and their association with efficient healthcare utilization has not been sufficiently studied in South Korea. One study conducted in 2015 investigated the proxy association by using hospice beds; however, this study could not address actual patients’ hospice service utilization due to the absence of data before the hospice policy initiation [22]. Therefore, the purpose of this study is to investigate how hospice service for lung cancer patients is associated with end-of-life healthcare utilization after policy implementation using Korean Nationwide Health Insurance (NHI) Claims data.

## 2. Materials and Methods

### 2.1. Study Population

**Figure 1:**
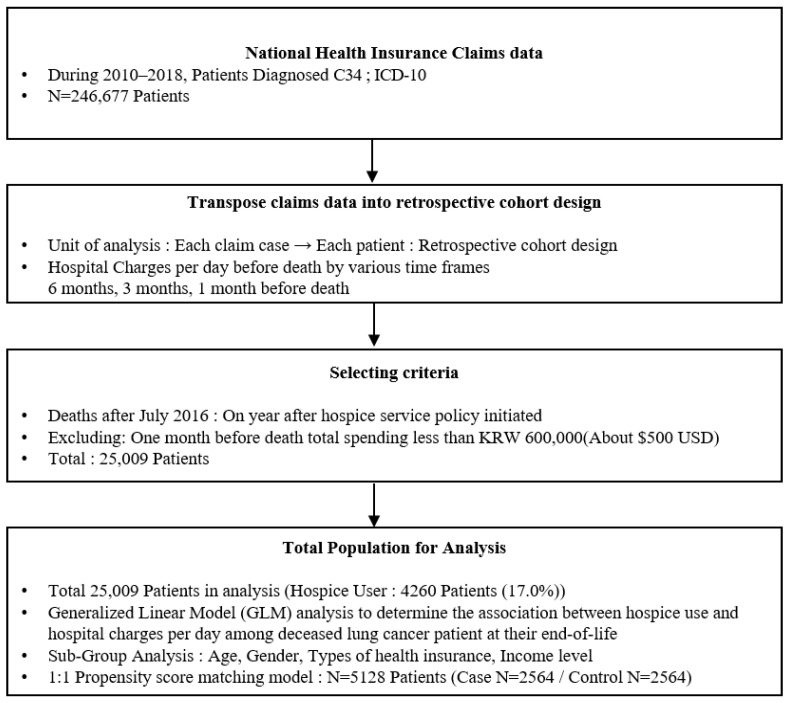
Flow chart of subject selection.

We used Korean National Health Insurance Service (NHIS) data from 2008–2018 that accounted for all lung cancer patients’ health insurance claims. We first identified individuals diagnosed with lung cancer according to the International Classification of Diseases, version 10 (ICD-10): C34. Then, we examined individuals newly diagnosed with lung cancer after 2010 by reviewing the two-year pre-diagnosis claims. Therefore, we used nationwide newly diagnosed lung cancer patients’ health insurance claims during 2010–2018, accounting for 246,677 patients. Subsequently, we transposed the dataset into a retrospective cohort design study with the unit of analysis being each lung cancer patients’ healthcare utilization. Next, we selected the study population as patients who died after July 2016 (1 year after hospice service policy was implemented), and we observed hospice users’ healthcare utilization before death by different periods through comparison with non-hospice service users. In order to identify actual lung cancer patients within the insurance claims dataset, we excluded patients whose one month before death total spending was less than KRW 600,000 (about USD 500 USD). Finally, we obtained a total sample for analysis of 25,009 lung cancer patients (Figure 1). For the model validation, a 1:1 propensity score matching method was used. To match the case group (hospice service users) with the control group (non-hospice service users), we used age, gender, health insurance type, income level, survival time, and diagnosed year. As a result, we identified a total sample of 5128 patients with a control group of *N* = 2564 and a case group of *N* = 2564 for the additional analysis.

### 2.2. Variables

The primary outcome was daily healthcare utilization, which was measured by hospital charges per day for deceased lung cancer patients according to different periods before death (1 month, 3 months, and 6 months before death). Hospital charges per day were defined as daily amounts of healthcare utilization for each lung cancer patient’s different before-death periods. We also identified whether each patient had the hospice service or was not within the claims dataset. Control variables included age, gender, type of health insurance, income level, year at diagnosis of lung cancer, and survival time. Patient age was categorized into the following groups (in years): 49 or younger, 50–59, 60–69, 70–79, and 80 or older. Types of health insurance were divided into medical aid beneficiaries, self-employed insured, and employee insured. We categorized income according to the NHI contribution quintile: the 1st quintile (the 20th or lower percentile), the 2nd quintile (the 21st–40th percentile), the 3rd quintile (the 41st–60th percentile), the 4th quintile (the 61st–80th percentile), and the 5th quintile (the 80th or higher percentile). The diagnosed year was from 2014 to 2018. Survival time was the time from lung cancer diagnosis to death measured by days.

### 2.3. Statistical Analyses

We first calculated the frequencies of categorical variables and performed χ2 tests to examine differences in each variable by hospice service use. Next, continuous variables including hospital charges per day (different periods before death) and survival times were examined, and we performed ANOVA tests to examine differences by hospice service use as well. Then, we employed Generalized Linear Model (GLM) analysis to determine the effect of hospice service use on healthcare charges per day in deceased lung cancer patients’ end of life. Additionally, subgroup analyses were performed to investigate the difference in healthcare utilization by use of hospice service depending on patient characteristics. Finally, the GLM model was also utilized for propensity score matched sample for model validation. Analyses were conducted in 2020–2021. This study’s type I error rate was set at 0.05 (two sided) for all statistical analyses. All statistical analyses were performed using SAS statistical software, version 9.4 (SAS Institute Inc., Cary, NC, USA).

## 3. Results

### 3.1. Patient Sample and Characteristics

The total number of lung cancer patients was 25,009 (hospice users: 4260 (17.0%)). Hospice use rate was high among the 49 years old or younger, female, employee-insured, and high-income groups and patients diagnosed during 2014–2016. Hospital charges per day were lower for hospice service users for each death-before period (Table 1).

### 3.2. Association between Hospice Service and Efficient Healthcare Utilization

With other variables adjusted, hospice users were associated with lower hospital charges per day at their end of life compared to non-users. Cost was lower by KRW 33,806 (about USD 31) for 1 month before death, KRW 25,948 (about USD 24) for 3 months before death, and KRW 20,620 (about USD 19) for 6 months before death (Table 2).

### 3.3. Subgroup Analysis and Propensity Score Matching Model

**Figure 2:**
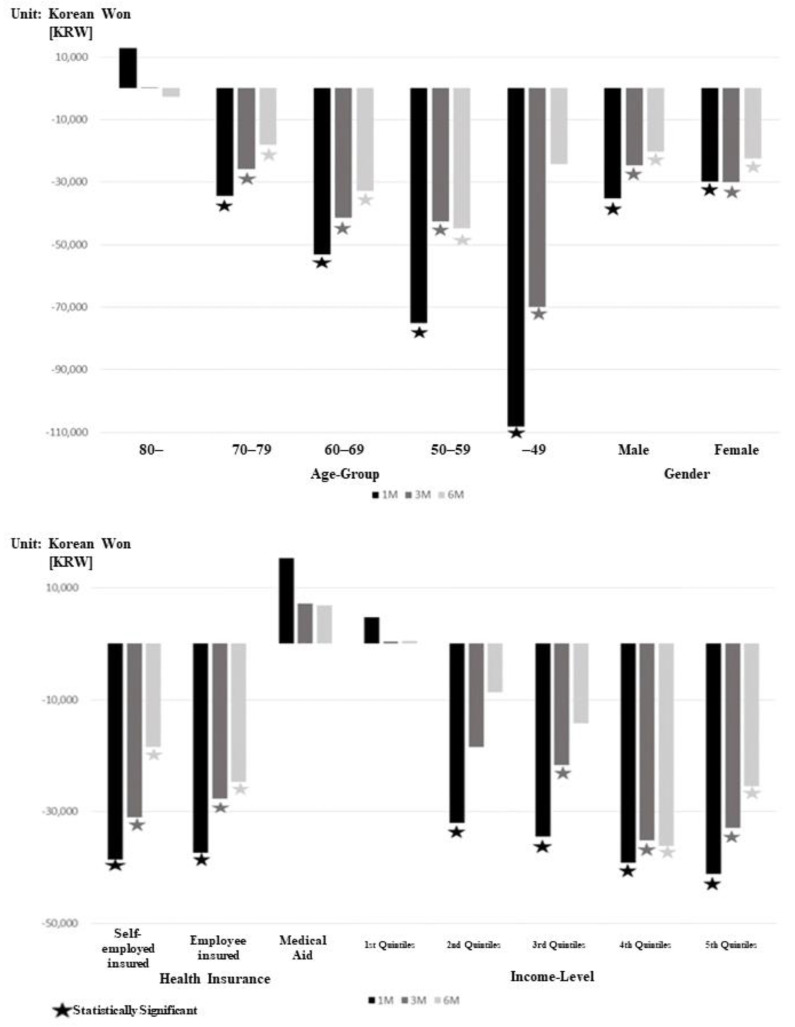
The result of subgroup analysis to investigate differences in hospital charges by subgroups based on the use of hospice services.

A similar trend appeared in the subgroup analysis in which hospice service users were associated with lower hospital charges per day among various patient characteristics, including age, gender, types of health insurance, and income level. Some characteristics including age 80+, medical aid beneficiary, and 1st quintile income group had slightly higher hospital charges per day; however, not all of it was statistically significant (Figure 2). The results of the propensity score matching model analysis are shown in Table 3, and the results also confirmed the full model that hospice use is associated with lower hospital charges per day among lung cancer patients at the end of life (Table 3).

## 4. Discussion

This study examined the association between hospice service use and lung cancer patients’ hospital charges per day at their end-of-life stage. The National Health Insurance claims data used in this study included all lung cancer patients enrolled in the NHI program (basically covering all the population) who died 1 year after hospice policy coverage was initiated. To compare healthcare utilization differences according to the periods before death, we used hospital charges per day within the last six months, three months, and one month before death.

We found that lung cancer patients with hospice service had lower hospital charges per day within the last months before death than deceased patients without hospice experience. Our results align with previous research findings that hospice and palliative care reduce healthcare costs for terminal lung cancer patients [23,24,25,26]. Unnecessarily aggressive end-of-life care does not cure the patient’s disease; instead, it increases healthcare costs and may lower the quality of life [27,28]. It has also been shown that hospice care reduces overly aggressive care and death outside of an acute care hospital [22,27,29,30,31,32,33,34]. Similar to a previous study conducted using a proxy measure (# of hospice beds) using a nationwide dataset in Korea [22], our study also highlighted evidence of efficient patient care for end-of-life lung cancer patients using the actual hospice utilization dataset. The results of our study deliver an essential message to the NHI program and policymakers that costly healthcare resources are associated with end-of-life treatment of lung cancer inpatients and may have no impact on extending life as compared to hospice care, despite significant hospital inpatient charge per day reductions found in some hospice-using patients. Hospice and palliative care can reduce the utilization of expensive acute care hospital resources and improve the quality of life for terminal cancer patients and their families as well [25,34]. However, further studies are required on the quality of hospice services for cancer patients and their families due to the expected increase in cancer patients in the aged Korean society.

Furthermore, we found that much lower hospital charges per day were associated with younger, male, health-insured, and relatively higher income groups using subgroup analysis. Considering the effectiveness and end-of-life efficiency in hospice care, it is not surprising that public or private investments in hospice care should be implemented for patients with terminal cancer [25,26,29,30,31,32,33,34]. However, many cancer patients continue with life-sustaining anti-cancer treatments that they may not need and that are not associated with more prolonged survival [26,35]. Therefore, a financially viable NHI program requires an expansion of hospice services nationwide within hospitals and in specialized hospice care facilities. Hospice services may reduce the overall lung cancer disease burden and provide a greater quality of life to end-of-life cancer patients [22]. Another strength of our study was using all nationwide lung cancer patients’ claims data with a retrospective design that contains all age groups, contributing to the robustness of our study. Finally, we used propensity score matched analysis for model validation. Using this methodology, the result using entire sample was likely validated, since patients who used hospice service and those who did not were different. This might reducing the impact of the existence of confounding factors.

This study had several limitations, and caution is required when interpreting the results and generalizing its findings. First, we analyzed National Health Insurance claims from all lung cancer patients who died across the country during a defined period. However, Korea’s unique insurance and healthcare delivery system may significantly limit the generalizability of the findings to other countries. This includes that healthcare utilization depends on the type of health insurance system and the provider’s ability to negotiate the price of healthcare services. Furthermore, it is possible that patient characteristics influenced the results because it was not a randomized controlled or cohort study between the two groups (hospice user vs. non-user). However, the research method used in this study was one of the best ways to evaluate the effectiveness of hospice service use in Korea, because there is not yet enough usage of hospice service using the health insurance claims dataset.

Furthermore, we investigated only lung cancer patients. Therefore, our results will differ from those regarding hospital charges per day and length of stay for patients with other types of cancer, possibly weakening the reliability of our findings. A lack of data to analyze important aspects of patient care quality was another limitation of our study. Finally, the data source of this study does not include cancer patients’ clinical information, which is one limitation of health insurance claims data. We employed survival time in the model; however, further study is required using cancer registry data or a cohort dataset controlling for proper severity. Despite these limitations, to the best of our knowledge, this is one of the only studies to analyze and explore healthcare utilization associated with hospice service use and provide meaningful results.

## 5. Conclusions

This study found lower end-of-life hospital charges per day among lung cancer patients who received hospice services near death. Furthermore, this finding was different regarding various patients’ socioeconomic characteristics. In the era of an aging society, efficient healthcare utilization in Korea and worldwide is an essential issue. Therefore, health policymakers and the National Health Insurance program need to consider expanding hospice services for terminal cancer patients, as hospices serve a growing need in an aged society.

## Figures and Tables

**Table 1:** General characteristics of study sample by hospice service use.

Variables	Total	Hospice Service				*p*-Value
		Non-User		User		
Total (*N*, %)	25,009	20,749 (83.0)		4260 (17.0)		
Age Group (*N*, %)						
80-	6285 (25.1)	5204 (82.8)		1081 (17.2)		<0.01
70–79	9184 (36.7)	7657 (83.4)		1527 (16.6)		
60–69	6195 (24.8)	5181 (83.6)		1014 (16.4)		
50–59	2616 (10.5)	2132 (81.5)		484 (18.5)		
−49	729 (2.9)	575 (78.9)		154 (21.1)		
Gender (*N*, %)						
Male	18,917 (75.6)	15,803 (83.5)		3114 (16.5)		<0.01
Female	6080 (24.4)	4936 (81.2)		1144 (18.8)		
Health Insurance (*N*, %)						
Self-employed insured	7300 (29.2)	6092 (83.5)		1208 (16.5)		<0.01
Employee insured	15,481 (61.9)	12,725 (82.2)		2756 (17.8)		
Medical Aid	2216 (8.9)	1922 (86.7)		294 (13.3)		
Income Level (*N*, %)						
1st Quintile	2603 (10.4)	2245 (86.2)		358 (13.8)		<0.01
2nd Quintile	4363 (17.4)	3625 (83.1)		738 (16.9)		
3rd Quintile	4170 (16.7)	3472 (83.3)		698 (16.7)		
4th Quintile	5400 (21.6)	4489 (83.1)		911 (16.9)		
5th Quintile	8473 (33.9)	6918 (81.6)		1555 (18.4)		
Diagnosed year (*N*, %)						
2014	1445 (5.8)	1122 (77.6)		323 (22.4)		<0.01
2015	2886 (11.5)	2278 (78.9)		608 (21.1)		
2016	7411 (29.6)	6087 (82.1)		1324 (17.9)		
2017	8405 (33.6)	7057 (84.0)		1348 (16.0)		
2018	4862 (19.4)	4205 (86.5)		657 (13.5)		
Survival Time (day) (Mean, SD)	323	311 (326)		382 (350)		<0.01
Hospital charges per day (1 M before death) (Mean, SD)	290,973	297,333 (312,473)		260,017 (122,071)		<0.01
Hospital charges per day (3 M before death) (Mean, SD)	268,738	274,031 (262,168)		242,977 (156,739)		<0.01
Hospital charges per day (6 M before death) (Mean, SD)	267,626	272,055 (244,318)		246,068 (158,531)		<0.01

**Table 2:** Result of GLM analysis: association between hospice service and hospital charges per day among deceased lung cancer patients at their end of life.

Variables	One Month Before Death Spending (KRW)	*p*-Value	Three Months Before Death Spending (KRW)	*p*-Value	Six Month Before Death Spending (KRW)	*p*-Value
Hospice Service						
Non-User	ref		ref		ref	
User	−33,806	<0.01	−25,948	<0.01	−20,620	<0.01
Age Group						
80-	ref		ref		ref	
70–79	50,833	<0.01	41,150	<0.01	43,586	<0.01
60–69	83,805	<0.01	72,689	<0.01	76,135	<0.01
50–59	113,926	<0.01	104,677	<0.01	109,322	<0.01
−49	148,995	<0.01	128,106	<0.01	132,039	<0.01
Gender						
Male	ref		ref		ref	
Female	10,094	0.02	9959	0.01	8216	0.02
Health Insurance						
Self-employed insured	38,082	0.02	40,289	<0.01	33,487	0.01
Employee insured	41,559	0.01	37,818	0.01	34,009	0.01
Medical aid	ref		ref		ref	
Income Level						
1st Quintile	ref		ref		ref	
2nd Quintile	−9626	0.53	−14,088	0.28	−10,186	0.41
3rd Quintile	−8151	0.60	−11,812	0.37	−8129	0.51
4th Quintile	−6513	0.67	−12,804	0.33	−7013	0.57
5th Quintile	−1951	0.90	−9788	0.45	−5114	0.67
Diagnosed year						
2014	ref		ref		ref	
2015	3935	0.69	−8729	0.30	−126	0.99
2016	11,145	0.31	−3397	0.72	3761	0.67
2017	43,760	<0.01	36,911	<0.01	46,396	<0.01
2018	82,069	<0.01	71,921	<0.01	77,092	<0.01
Survival Time (days)	−7.8	0.40	−24.3	<0.01	−28.7	<0.01

**Table 3:** Result of GLM analysis for propensity score matched sample: association between hospice service and hospital charges per day among deceased lung cancer patients at their end of life.

Variables	One Month Before Death Spending (KRW)	*p*-Value	Three Month Before Death Spending (KRW)	*p*-Value	Six Month Before Death Spending (KRW)	*p*-Value
Hospice Service						
Non-User	ref		ref		ref	
User	−18,178	<0.01	−9577	0.04	−4732	0.03
Age Group						
80–	ref		ref		ref	
70–79	32,581	<0.01	30,706	<0.01	33,992	<0.01
60–69	64,879	<0.01	54,407	<0.01	56,818	<0.01
50–59	86,492	<0.01	94,790	<0.01	84,199	<0.01
−49	87,369	<0.01	84,213	<0.01	76,009	<0.01
Gender						
Male	ref		ref		ref	
Female	22,619	<0.01	19,275	<0.01	13,400	0.04
Health Insurance						
Self-employed insured	17,212	0.50	24,767	0.26	31,141	0.15
Employee insured	20,219	0.42	25,582	0.23	32,859	0.11
Medical Aid	ref		ref		ref	
Income Level						
1st Quintile	ref		ref		ref	
2nd Quintile	−5379	0.82	−5072	0.80	−11,698	0.56
3rd Quintile	−1198	0.96	−3406	0.87	−5417	0.79
4th Quintile	−8805	0.71	−9707	0.63	−17,565	0.38
5th Quintile	−4528	0.85	−8200	0.68	−14,298	0.47
Diagnosed year						
2014	ref		ref		ref	
2015	−11,487	0.63	−4007	0.85	21,118	0.29
2016	9622	0.70	−665	0.98	15,805	0.45
2017	29,505	0.26	29,481	0.18	46,727	0.03
2018	63,641	0.02	57,447	0.01	74,933	<0.01
Survival Time (days)	−3.7	0.84	−36.0	0.02	−27.3	0.07

## Data Availability

The data presented in this study are available on request from the corresponding author. All data generated or analysed during this study are included in this published article.

## References

[1] Fitzmaurice C., Allen C., Barber R.M., Barregard L., Bhutta Z.A., Brenner H., Dicker D.J., Chimed-Orchir O., Dandona R., Global Burden of Disease Cancer Collaboration (2017). Global, Regional, and National Cancer Incidence, Mortality, Years of Life Lost, Years Lived with Disability, and Disability-Adjusted Life-years for 32 Cancer Groups, 1990 to 2015: A Systematic Analysis for the Global Burden of Disease Study. JAMA Oncol..

[2] Statistics Korea (2018). Cause of Death Statistics in Korea.

[3] Park J.H., Lee K.S., Choi K.S. (2013). Burden of cancer in Korea during 2000–2020. Cancer Epidemiol..

[4] Meropol N.J., Schulman K.A. (2007). Cost of cancer care: Issues and implications. J. Clin. Oncol..

[5] Sullivan R., Peppercorn J., Sikora K., Zalcberg J., Meropol N.J., Amir E., Khayat D., Boyle P., Autier P., Tannock I.F. (2011). Delivering affordable cancer care in high-income countries. Lancet Oncol..

[6] DiMasi J.A., Hansen R.W., Grabowski H.G. (2003). The price of innovation: New estimates of drug development costs. J. Health Econ..

[7] McCall N. (1984). Utilization and costs of Medicare services by beneficiaries in their last year of life. Med. Care.

[8] Lubitz J.D., Riley G.F. (1993). Trends in Medicare payments in the last year of life. N. Engl. J. Med..

[9] Brown M.L., Riley G.F., Schussler N., Etzioni R. (2002). Estimating health care costs related to cancer treatment from SEER-Medicare data. Med. Care.

[10] Yabroff K.R., Lamont E.B., Mariotto A., Warren J.L., Topor M., Meekins A., Brown M.L. (2008). Cost of care for elderly cancer patients in the United States. J. Natl. Cancer Inst..

[11] Gardiner C., Brereton L., Frey R., Wilkinson-Meyers L., Gott M. (2014). Exploring the financial impact of caring for family members receiving palliative and end-of-life care: A systematic review of the literature. Palliat. Med..

[12] Beccaro M., Costantini M., Giorgi Rossi P., Miccinesi G., Grimaldi M., Bruzzi P., ISDOC Study Group (2006). Actual and preferred place of death of cancer patients. Results from the Italian survey of the dying of cancer (ISDOC). J. Epidemiol. Community Health.

[13] World Health Organization (2014). Global Atlas of Palliative Care at the End of Life.

[14] Rome R.B., Luminais H.H., Bourgeois D.A., Blais C.M. (2011). The role of palliative care at the end of life. Ochsner. J..

[15] Park B.K., Park Y.M., Kim Y.S., Hong S.J., Park S.C., Kim Y.N., Yook T.M., Bae S.J. (2019). Analysis of the Use and Effect of Inpatient Hospice in Terminal Cancer Patients after Applying Health Insurance, Research Report 2018-20-028.

[16] Health Insurance Review and Assessment Service (2015). Application of Palliative Care Health Insurance—Medical Care Benefit Cost and Practical Guidance.

[17] National Cancer Center of Korea (2018). Status of Hospice Palliative Care 2016.

[18] National Cancer Center of Korea (2019). Status of Hospice Palliative Care 2017.

[19] Taylor D.H. (2009). The effect of hospice on Medicare and informal care costs: The U.S. Experience. J. Pain Symptom Manag..

[20] Higginson I.J., Finlay I., Goodwin D.M., Cook A.M., Hood K., Edwards A.G.K., Douglas H.-R., Norman C.E. (2002). Do hospital-based palliative teams improve care for patients or families at the end of life?. J. Pain Symptom Manag..

[21] Zhang B., Wright A.A., Huskamp H.A., Nilsson M.E., Maciejewski M.L., Earle C.C., Block S.D., Maciejewski P.K., Prigerson H.G. (2009). Health care costs in the last week of life: Associations with end-of-life conversations. Arch. Intern. Med..

[22] Kim S.J., Han K.T., Kim T.H., Park E.C. (2015). Does hospital need more hospice beds? Hospital charges and length of stays by lung cancer inpatients at their end of life: A retrospective cohort design of 2002–2012. Palliat. Med..

[23] Huo J., Hong Y.R., Turner K., Bian J., Grewal R., Wilkie D.J. (2019). Utilization pattern and service settings of palliative care for patients with metastatic non-small cell lung cancer. Cancer.

[24] Hwang J., Shen J., Kim S.J., Chun S.Y., Kioka M., Sheraz F., Kim P., Byun D., Yoo J.W. (2019). Ten-Year Trends of Utilization of Palliative Care Services and Life-Sustaining Treatments and Hospital Costs Associated With Patients With Terminally Ill Lung Cancer in the United States From 2005 to 2014. Am. J. Hosp. Palliat. Care.

[25] Triplett D.P., LeBrett W.G., Bryant A.K., Bruggeman A.R., Matsuno R.K., Hwang L., Boero I.J., Roeland E.J., Yeung H.N., Murphy J.D. (2017). Effect of Palliative Care on Aggressiveness of End-of-Life Care Among Patients With Advanced Cancer. J. Oncol. Pract..

[26] Mor V., Wagner T.H., Levy C., Ersek M., Miller S.C., Gidwani-Marszowski R., Joyce N., Faricy-Anderson K., Corneau E.A., Lorenz K. (2019). Association of Expanded VA Hospice Care With Aggressive Care and Cost for Veterans With Advanced Lung Cancer. JAMA Oncol..

[27] Ho T.H., Barbera L., Saskin R., Lu H., Neville B.A., Earle C.C. (2011). Trends in the aggressiveness of end-of-life cancer care in the universal health care system of Ontario, Canada. J. Clin. Oncol..

[28] Langton J.M., Blanch B., Drew A.K., Haas M., Ingham J.M., Pearson S.A. (2014). Retrospective studies of end-of-life resource utilization and costs in cancer care using health administrative data: A systematic review. Palliat. Med..

[29] Obermeyer Z., Makar M., Abujaber S., Dominici F., Block S., Cutler D.M. (2014). Association between the Medicare hospice benefit and health care utilization and costs for patients with poor-prognosis cancer. JAMA.

[30] Chiang J.K., Kao Y.H., Lai N.S. (2015). The Impact of Hospice Care on Survival and Healthcare Costs for Patients with Lung Cancer: A National Longitudinal Population-Based Study in Taiwan. PLoS ONE.

[31] Earle C.C., Landrum M.B., Souza J.M., Neville B.A., Weeks J.C., Ayanian J.Z. (2008). Aggressiveness of cancer care near the end of life: Is it a quality-of-care issue?. J. Clin. Oncol..

[32] Wang J.P., Wu C.Y., Hwang I.H., Kao C.H., Hung Y.P., Hwang S.J., Li C.-P. (2016). How different is the care of terminal pancreatic cancer patients in inpatient palliative care units and acute hospital wards? A nationwide population-based study. BMC Palliat. Care.

[33] Merchant S.J., Brogly S.B., Goldie C., Booth C.M., Nanji S., Patel S.V., Lajkosz K., Baxter N.N. (2018). Palliative Care is Associated with Reduced Aggressive End-of-Life Care in Patients with Gastrointestinal Cancer. Ann. Surg. Oncol..

[34] Davies E., Higginson I.J., World Health Organization (2004). Better Palliative Care for Older People.

[35] Mohammed A.A., Al-Zahrani O., Salem R.A., Elsayed F.M. (2019). Aggressive Care at the End of Life; Where Are We?. Indian J. Palliat. Care.

